# Nonadditive gene expression contributing to heterosis in partially heterozygous maize hybrids is predominantly regulated from heterozygous regions

**DOI:** 10.1111/nph.70128

**Published:** 2025-04-07

**Authors:** Marion Pitz, Jutta A. Baldauf, Hans‐Peter Piepho, Frank Hochholdinger

**Affiliations:** ^1^ Institute of Crop Science and Resource Conservation, Crop Functional Genomics University of Bonn 53113 Bonn Germany; ^2^ Biostatistics Unit, Institute of Crop Science University of Hohenheim 70599 Stuttgart Germany

**Keywords:** expression quantitative trait loci, heterosis, hybrid, maize, recombinant inbred line, Transcriptome Wide Association Study

## Abstract

- Hybrids often perform better than their homozygous parents, a phenomenon that is commonly referred to as heterosis. Heterosis is widely utilized in modern agriculture, although its molecular basis is not very well understood.
- In this study, we backcrossed an intermated recombinant inbred line population of maize (*Zea mays* L.) with its parental inbred lines B73 and Mo17. The resulting hybrids exhibited different degrees of heterozygosity and heterosis. We identified nonadditively expressed genes, which are expressed differently from their mid‐parental level. In addition, we surveyed their regulation by investigating expression quantitative trait loci (eQTL).
- Nonadditively expressed genes explain up to 27% of heterotic variance in the backcross hybrids. Furthermore, nonadditively expressed genes are regulated almost exclusively from heterozygous regions of the genome. We observed that nonadditive expression patterns are distinctly regulated depending on the genetic origin of the higher expressed parent. As a consequence, these regulatory regimes lead to higher gene activity in most nonadditively expressed genes in the hybrids.
- We demonstrated that nonadditive expression patterns contribute to heterosis and their mode of regulation might translate phylogenetic distance into vigorous hybrids. Based on our results, we hypothesize that diverging regulatory preferences in inbred lines are beneficial for selecting parental combinations for hybrid breeding.

## Introduction

The term heterosis describes the observation that hybrid progeny of genetically distinct parents display superior agricultural performance (Shull, 1914). The introduction of hybrids in maize breeding in the 1930s is considered one of the landmark innovations of modern agriculture and has contributed to an enormous increase in yield (Duvick, 2005; Hochholdinger & Baldauf, 2018; Hochholdinger & Yu, 2024). It has been observed that the phylogenetic distance between the parental inbred lines is positively associated with heterosis (East, 1936). The observation that specific parent combinations result in especially high levels of heterosis has resulted in the definition of typical female and male heterotic groups (Reif *et al*., 2005). Other crops, such as rice, also benefit from the classification of genotypes into heterotic groups and their combination as heterotic patterns (Melchinger & Gumber, 1998; Beukert *et al*., 2017).

Heterosis is observed in all parts of the plant throughout development, but is typically investigated for aboveground traits related to yield (Paril *et al*., 2024). In maize roots, which play an important role in the overall performance of plants, heterosis becomes apparent 5–7 d after germination (Hoecker *et al*., 2006).

Classical genetic concepts to explain heterosis include the dominance and overdominance models. The dominance model postulates that heterosis is caused by complementation of slightly deleterious alleles at many loci in the hybrid by dominant or at least stronger alleles (Jones, 1917). The overdominance model postulates that two different alleles at the same locus cause heterosis by their interaction and that the heterozygous state itself is advantageous to the homozygous situation of the parents (East, 1936). Despite examples of single genes displaying overdominance (Krieger *et al*., 2010; Lin *et al*., 2020), none of these models alone can fully explain heterosis (Duvick, 2001; Chen & Birchler, 2013; Hochholdinger & Yu, 2024).

Genes with differential expression between two maize lines can show a variety of expression levels in the resulting hybrid. They can display additive expression, reflecting the average expression of their parents, or deviate from this pattern and display nonadditive expression (Hochholdinger & Hoecker, 2007). Depending on the surveyed tissues, developmental stages, and genotypes, maize displays a highly variable degree of nonadditive gene expression (Uzarowska *et al*., 2007; Hoecker *et al*., 2008; Paschold *et al*., 2012; Zhang *et al*., 2016). Reciprocal maize hybrids of B73 and Mo17 generally share the same nonadditive pattern (Stupar & Springer, 2006; Baldauf *et al*., 2016). In early primary roots and developing ear shoots of the same hybrids, a trend toward adoption of high parent expression, rather than low parent expression, was observed (Paschold *et al*., 2012; Qin *et al*., 2013; Baldauf *et al*., 2016), but not further investigated in detail. Both additive and nonadditive expression have been considered to contribute to heterosis (Guo *et al*., 2006; Stupar & Springer, 2006; Hoecker *et al*., 2008; Stupar *et al*., 2008; Baldauf *et al*., 2016). The observation that nonadditive genes are conserved under stress conditions and mostly belong to evolutionarily less conserved, nonsyntenic genes suggests that they are involved in adaptation to different environments or stress conditions (Baldauf *et al*., 2016; Marcon *et al*., 2017).

Gene expression differences are the result of alterations in gene regulation. Regulatory elements can be classified as *cis* if they are positioned close to the regulated gene, and *trans* if the element is located at a different position, often on a different chromosome (Jansen & Nap, 2001). A possible connection between transcriptional variation in the regulation of *cis*‐ and *trans*‐acting factors and hybrid performance was discussed (Botet & Keurentjes, 2020) and an association of *trans*‐regulated gene expression in hybrids with paternal alleles was shown in maize (Swanson‐Wagner *et al*., 2009).

Recombinant inbred line (RIL) populations as well as backcross populations have been extensively used in genetics for quantitative trait locus (QTL) mapping, candidate gene identification, and heterosis studies (Rahman *et al*., 2011; Pan *et al*., 2017; Huo *et al*., 2023; Yang *et al*., 2024). They can further be used to identify expression quantitative trait loci (eQTL). These are genomic regions associated with variation in gene expression across the mapping population and provide direct insights into the regulation of gene expression (Jansen & Nap, 2001).

In this study, we analyzed the transcriptomes of the maize intermated B73 and Mo17 (IBM) RIL Syn. 4 population (Lee *et al*., 2002) and their partially homozygous and heterozygous backcross hybrid populations with the original parents B73 and Mo17 (Supporting Information Fig. S1). We demonstrated that nonadditive gene expression patterns influence the manifestation of heterosis in seedling root development. We further showed that regulatory elements of nonadditive genes are predominantly located in heterozygous regions, suggesting that heterozygosity at the regulatory level promotes a higher expression in the hybrid compared to the parental average. Depending on their parental genetic origin, these regulatory elements act predominantly in either *cis* or *trans*, possibly influencing the formation of heterotic patterns.

## Materials and Methods

### Plant material

For our study, we backcrossed a subset of 112 IBM‐RILs of the maize (*Zea mays* L.) intermated RIL population (IBM‐RIL Syn. 4; Lee *et al*., 2002) as males to their original parents B73 and Mo17 as females. The IBM‐RIL Syn. 4 population was generated by crossing the maize inbred lines B73 and Mo17, followed by four generations of intercrossing and subsequent self‐pollination of their progeny. Different IBM‐RILs are highly homozygous and diverse regarding their genomic regions contributed by B73 and Mo17. B73 × IBM‐RIL and Mo17 × IBM‐RIL backcross hybrids are partially homozygous and heterozygous. Backcross hybrids of a specific IBM‐RIL show contrasting homozygous and heterozygous genomic regions. We additionally included the parental inbred lines B73 and Mo17, as well as their reciprocal hybrids B73 × Mo17 and Mo17 × B73 as fully heterozygous reference hybrids.

### Experimental design and harvesting of plant material

We germinated all genotypes in an alpha‐design with incomplete blocks, as described in detail in Pitz *et al*. (2024), containing three biological replicates of each of the 112 IBM‐RILs (336 samples) and each of the 112 backcross hybrids, B73 × IBM‐RIL and Mo17 × IBM‐RIL (672 samples). Moreover, we included 48 biological replicates of the maternal inbred lines B73 and Mo17 and 24 biological replicates of the reciprocal hybrids B73 × Mo17 and Mo17 × B73 as reference hybrids. In total, we analyzed 1152 samples. For each sample, we sterilized 25 kernels with 10% H_2_O_2_, pre‐germinated them in germination paper rolls, and placed them in distilled water in a climate chamber with 16 h : 8 h; 26°C : 21°C; light : dark period. After 3 d, we selected up to eight seedlings per sample based on similar primary root length, and placed them into an aeroponic growth system (‘Elite Klone Machine 96’; TurboKlone, Sparks, NV, USA). After another 4 d, we collected the distal part of the primary root, which included the root tip as well as the meristematic and elongation zone. We stored the roots immediately in liquid nitrogen until RNA extraction (Pitz *et al*., 2024).

### RNA‐sequencing processing and single nucleotide polymorphism (SNP) calling

We ground each sample, consisting of up to eight primary roots of the same genotype, in liquid nitrogen before RNA extraction with the RNeasy Plant Mini Kit (Qiagen). The next generation sequencing core facility in Bonn, Germany (https://btc.uni‐bonn.de/ngs/), assessed the RNA quality, using a Bioanalyzer (RNA ScreenTape + TapeStation Analysis Software 3.2; Agilent Technologies, Santa Clara, CA, USA), constructed the cDNA libraries necessary for RNA‐sequencing, following the protocol for TruSeq reversely‐stranded mRNA (Illumina, San Diego, CA, USA), and sequenced 100‐bp paired‐end reads on a NovaSeq 6000 S4 flow cell machine (Illumina). We trimmed and filtered the raw‐reads with Trimmomatic (v.0.39) in paired‐end mode with the following settings: ILLUMINACLIP:adapters/TruSeq3‐PE‐2.fa:2:30:10:8:True, LEADING:3, TRAILING:3, MAXINFO:30:0.8, and MINLEN:40 (Bolger *et al*., 2014). We aligned the trimmed reads to the maize reference genome (B73v5, ftp.ensemblgenomes.org/pub/plants/release‐52/fasta/zea_mays/dna/Zea_mays.Zm‐B73‐REFERENCE‐NAM‐5.0.dna.toplevel.fa.gz) after indexing (exon information from http://ftp.ensemblgenomes.org/pub/plants/release‐52/gff3/zea_mays/Zea_mays.Zm‐B73‐REFERENCE‐NAM‐5.0.52.gff3.gz) with Hisat2 (v.2.2.1; Kim *et al*., 2015) with the settings: ‐q ‐‐phred 33 ‐‐rna‐strandedness RF ‐‐min‐intronlen 20 ‐‐max‐intronlen 60 000. We then used samtools from htslib (v.1.14) (Danecek *et al*., 2021) and Picard tools (v.2.27.1; http://broadinstitute.github.io/picard/) for formatting and duplicate removal. We then counted uniquely mapped reads with htseq‐count (v.2.0.1) (Anders *et al*., 2015) and excluded samples with a library size < 5 million counted reads.

We prepared the alignments for SNP calling with GATK's HaplotypeCaller by adding read group information (Picard AddOrReplaceReadGroups, v.2.27.1), filtering for uniquely mapped reads (mapping quality ≥ 60) and formatting (Samtools view, v.1.14). Subsequently, we split alignments at positions with N in the CIGAR files, for example intron‐spanning reads, by using GATK's SplitNCigarReads (v.2.4.6.1) (van der Auwera & O'Connor, 2020; GATK, 2023).

We performed SNP calling in two steps (Pitz *et al*., 2024). In brief, we performed a first SNP calling, based on which we excluded samples with low homozygosity within regions that should be homozygous or where genotypes in the hybrids did not match the parental genotypes. We identified SNPs between our Mo17 inbred line samples and the B73v5 reference genome. Additionally, we identified SNPs between the B73 inbred line samples and the B73v5 reference genome to exclude those loci where both the B73 and Mo17 lines show a nonreference allele. We then counted the frequency of the B73 and Mo17 alleles at each SNP locus in each sample (Vedder, 2024, adapted from Baldauf *et al*., 2020). First, we calculated the ratio of homozygous loci in the parental inbred lines. In case of < 95% homozygous loci, we excluded the sample. The same approach was followed for the homozygous regions of the partially homozygous backcross hybrids. Thus, we excluded 175 samples. Additionally, 10 samples had a library size of < 5 million read counts, and 17 samples were excluded because they were the only remaining replicate of a genotype. We then generated a variant call file (VCF) of known SNPs to be used in the second SNP calling. Subsequently, we excluded 90 hybrid samples because the paternal inbred was excluded, and eight IBM‐RIL paternal inbred lines because all corresponding hybrids were excluded. This left 852 samples for the second SNP calling. For this, we filtered the VCFs from the first SNP calling (https://gatk.broadinstitute.org/hc/en‐us/articles/360035890471‐Hard‐filtering‐germline‐short‐variants, accessed 18 December 2023) and used them for recalibration of base qualities with GATK's BaseRecalibrator and ApplyBSQR in the samples (https://gatk.broadinstitute.org/hc/en‐us/articles/360035531192‐RNAseq‐short‐variant‐discovery‐SNPs‐Indels‐, accessed 14 December 2023). We then used the HaplotypeCaller in BP_RESOLUTION mode. To subsequently eliminate positions without information, we filtered the result for positions with a coverage (DP) of ≥ 1. We obtained one database per triplet (IBM‐RIL, B73 × IBM‐RIL, Mo17 × IBM‐RIL) by combining the respective samples. To ensure genotyping of all possible SNP loci (not just thosepresent in the respective triplet), we added the B73 and Mo17 samples to each triplet's database. We filtered the resulting genotyped loci for SNPs with QD > 2, SOR < 3, MQ > 40, QUAL > 30, and FS < 60 using bcftools (v.1.17). To determine a list of high‐confidence B73 vs Mo17 SNPs, we confirmed the B73 allele in 90% or more of genotyped B73 samples (with at least 3 samples) and the Mo17 allele in 90% or more genotyped Mo17 samples (with at least 3 samples), all with high (> 10) GenotypeCall quality (GQ). Additionally, we identified SNPs that were not present in any B73 or Mo17 samples and were specific to the IBM‐RIL (homozygous or heterozygous and regardless of GQ).

### Classification of genomic regions and genes, determination of heterozygosity

We used the filtered SNP data (GQ > 10) to classify each IBM‐RIL genome into B73 or Mo17 regions and to mask regions which were not B73 or Mo17. In short, we implemented a distance function to group IBM‐RIL specific loci with a distance of < 2.5 Mbp into blocks. Blocks of at least 10 IBM‐RIL‐specific loci were masked as third origin regions. We then used a sliding window approach of 15 consecutive loci to eliminate singular loci that did not match their surrounding loci (Huang *et al*., 2009). We used the previously mentioned distance function to group loci carrying the same allele and located < 0.5 Mbp apart as a block, and all blocks were retained. We excluded two IBM‐RILs, which had > 50% of their genomes consisting of IBM‐RIL‐specific regions from a third parental origin and their respective hybrids (details in Pitz *et al*., 2024) Thus, 834 samples remained for final analyses. We filtered the dataset of each triplet to only include loci within the B73 or Mo17 regions of the IBM‐RILs and within exons of protein‐coding genes. We classified the genes as within a B73 region, within a Mo17 region, an unclassified region without SNP information, or masked them as IBM‐RIL specific. This verification was performed for each IBM‐RIL separately. We calculated the proportion of heterozygous to homozygous regions for each backcross hybrid by dividing the total lengths of classified heterozygous regions (B73 regions of the IBM‐RIL for the Mo17 × IBM‐RIL and Mo17 regions of the IBM‐RIL for B73 × IBM‐RIL) by the total lengths of all classified regions (not considering IBM‐RIL specific masked regions and regions without SNP information) (Dataset S1).

### Determination of differential and nonadditive gene expression patterns

We obtained differentially expressed genes by processing raw read counts with the Bioconductor package limma (v.3.50.3; Ritchie *et al*., 2015) in R (v.4.1.1). For each triplet combination composed of both parents and their hybrid offspring, only genes that are active in at least one of the three genotypes were considered In addition, we filtered lowly expressed genes by the filterByExpr() function of the Bioconductor package edgeR (v.3.36.0). We used the function CalcNormFactors() of limma to calculate normalization factors of the raw counts, which were later used by the voomWithQualityWeights() function of limma to obtain sample‐ and gene‐specific weights. We implemented the following model to estimate the gene expression across samples and genes (Law *et al*., 2014; Ritchie *et al*., 2015):

(Eqn 1) $$Y_{\text{jkln}\left(i\right)}=g_{i}+p_{k}+s_{l}+e_{\text{jkln}\left(i\right)}$$

We modeled the expression value of a specific gene of the respective genotype *i* as $\;Y_{\mathrm{jkl}\left(i\right)}$. The fixed effect for genotype *i* was represented by $g_{i}$. The remaining terms correspond to the experimental design, where the fixed effect for batch *k* nested within block *j* was included by $p_{k}$ and represents an incomplete block within a replicate. The random effect for system *l* nested within batch *k* and block *j* was included as $s_{l}$ and represents one of eight growth systems within each block. The random error effect for row *n* of genotype *i* in block *j* and batch *k* and system *l* was represented by $e_{\text{jkln}\left(i\right)}$. We subjected the fixed effects to the function voomWithQualityWeights() of limma, with $s_{l}$ serving as a block to obtain consolidated weights for library size and heterogeneity in sample quality as well as observational variance (Liu *et al*., 2015). Since the limma package does not provide a framework for random effects, we used limma's duplicateCorrelation() function to approximate the effect, with $s_{l}$ as a block (Smyth *et al*., 2005). Both the voomWithQualityWeights() and duplicateCorrelation() function were run twice, and we updated the resulting consensus value and used it in the lmFit() function. We made two contrasts for detecting differential expression: First, we compared each hybrid value to the mean of both parents and second, we compared both parents to each other using the contrast.fit() function. We computed moderated *t*‐statistics using an empirical Bayes method by the eBayes() function. We considered genes differentially expressed if they had an absolute log_2_FC > 1 and an adjusted *P*‐value ≤ 0.05 (Benjamini & Hochberg, 1995; Phipson *et al*., 2016).

### Determination of the contribution of nonadditive expression pattern to heterotic variance

We quantified the contribution of nonadditively expressed genes with higher than mid parent value (MPV) expression to heterosis. Therefore, we calculated the total heterotic variance, accounting for experimental factors and parental effects (*σ* ^2^_G_), using a linear mixed model (Eqn 2). By extending the model and including the numbers of nonadditive pattern genes (*σ* ^2^_Het_), we were able to calculate the proportion of variance attributable to nonadditive genes (p_Het_) (Eqn 3). This approach was adapted from the evaluation of single‐parent expression (SPE) contribution to heterosis from Pitz *et al*. (2024). We performed separate analyses for the B73 × IBM‐RIL and Mo17 × IBM‐RIL population. The phenotypic data for ‘total root length’, ‘total root volume’ and ‘total number of root tips’ were obtained from image analysis of the whole root system of each plant. ‘Lateral root density’ was obtained by counting the number of lateral roots emerged from the proximal first centimeter of the primary root (Pitz *et al*., 2024). We square‐root transformed the values of the traits ‘total root length’, ‘total root volume’, and ‘total number of root tips’, to fulfil the modeling assumptions.

The base model in each population for calculating *σ* ^2^_G_ was fitted as follows. We defined covariates to differentiate the IBM‐RILs and B73 and Mo17 as parental genotypes from their hybrids, but included them in the model simultaneously. We set the covariates initially to zero, but for observations corresponding to a parental genotype, we set the covariate for this genotype to 1. We set the parental covariates of both parents to 0.5 for observations on the hybrid. Thus, ultimately the terms $\beta_{1}x_{i1}+\ldots +\beta_{q}x_{\mathrm{iq}}$ model the general parental performance as well as the hybrid mid‐parent values (MPV). To adjust for the potential problem of rank deficiency in the design matrix for fixed effects, we removed the intercept and included dummy variables for replicates 2 and 3, which were included as fixed effects *b*_j_ (*j* = 2, 3). As the genetic variance of heterosis is likely not distributed around a mean of zero, we included the covariate *z*_i_ (defined below) via a fixed effect ϕ, so that the random heterosis effects will be distributed around the non‐zero mean ϕ.

(Eqn 2) $$Y_{\text{jklmnp}\left(i\right)}=\begin{array}{l} \beta_{1}x_{i1}+\ldots +\beta_{q}x_{\mathrm{iq}}+b_{j}+\phi z_{i}+g_{i}z_{i}+p_{k}+s_{l}\\ +\;t_{m}+r_{n}+e_{\text{jklmnp}\left(i\right)} \end{array}$$

We defined $Y_{\text{jklmnp}\left(i\right)}$ as the parental effect on MPH of the respective hybrid genotype *i* for a phenotypic trait. We defined $\;x_{\mathrm{iq}}$ as the parental covariables of parent *q* for genotype *i*, and $b_{j}$ as the fixed effect for block *j*. Further, we included $z_{i}*g_{i}$ as random effect of genotype *i*, whereas *z*_*i*_ is a dummy variable and set to *z*_*i*_ = 0 for parents and *z*_*i*_ = 1 for hybrids (Piepho *et al*., 2006). We defined $b_{j}$ as the fixed effect for block *j*, which represents the three complete replicates. This was realized via two dummy variables as described above, effectively setting *b*_1_ = 0. We added $p_{k}$ as random effect for batch *k* nested within block *j* and $s_{l}$ as the random effect for system *l* nested within batch *k* and block *j*, which represents one of eight growth systems within each batch. $t_{m}$ represents the random effect for one of four triplets *m* nested within system *l*, batch *k* and block *j*, which each consisted of an IBM‐RIL and both corresponding backcrosses or the reference inbreds B73 and Mo17 and a reciprocal hybrid. We let $r_{n}$ represent the random effect for a row of plants with the same genotype *n* nested within triplet *m*, system *l*, batch *k*, and block *j*. The random error effect $e_{\text{jklmnp}\left(i\right)}$ corresponds to plant *p* of genotype *i* in block *j*, batch *k*, system *l*, triplet *m*, and row *n*.

Next, we included the numbers of nonadditively expressed genes in the model. It should be noted that each pattern (1–4 in B73 × IBM‐RILs, 5–8 in Mo17 × IBM‐RILs) was represented by its own covariate. The numbers of nonadditive genes were set to 0 for parental genotypes.

(Eqn 3) $$Y_{\text{jklmnp}\left(i\right)}=\begin{array}{l} \beta_{1}x_{i1}+\ldots +\beta_{q}x_{\mathrm{iq}}+b_{j}+\gamma_{1}{\mathrm{sa}}_{i}+\gamma_{2}{\mathrm{sb}}_{i}\\ +\;\gamma_{3}{\mathrm{sc}}_{i}+\gamma_{4}{\mathrm{sd}}_{i}+\phi z_{i}+g_{i}z_{i}+p_{k}+s_{l}+t_{m}+r_{n}\\ +\;e_{\text{jklmnp}\left(i\right)} \end{array}$$

Thus, ${\mathrm{sa}}_{i}$, ${\mathrm{sb}}_{i},\;{\mathrm{sc}}_{i}$, and ${\mathrm{sd}}_{i}$ represent the covariables for nonadditively expressed genes in pattern 1 (${\mathrm{sa}}_{i}$) − 4 (${\mathrm{sd}}_{i}$) or 5 (${\mathrm{sa}}_{i}$) − 8 (${\mathrm{sd}}_{i}$) for hybrid *i*. We used the lme4 package (v.1.1–29) within R (v.4.0.1) for this analysis. [Correction added on 23 May 2025, after first online publication: details of some of the variables and other elements of the model described in the preceding paragraphs, and in Eqns 2 & 3, have now been updated.]

### eQTL analysis

We performed an eQTL analysis with the R/qtl2 package (v.0.22) (Broman *et al*., 2019) to identify positions that were significantly associated with gene expression values based on the masked and filtered SNP data as described in detail in Pitz *et al*. (2024). For each of the three cross‐types (IBM‐RIL, B73 × RILs, Mo17 × RILs), we took the classified and filtered SNP loci within B73 or Mo17 regions in the IBM‐RILs as marker input data. We used the positions of these SNP loci as preliminary genomic positions, as well as physical positions. As the phenotype data input in R/qtl2, we used the estimated expression means obtained from the model coefficients within the differential expression analysis of each genotype and gene. We removed samples with > 19% missing genotypes, duplicated genotypes, and markers with > 60% missing genotype information. We estimated the genetic map from the physical positions and genotype information and retained only the markers that were ≥ 1 cM apart to avoid an excess of redundant markers. We used a hidden Markov model and Haley–Knott regression (Haley & Knott, 1992) to establish the association between genotype and expression phenotype with a linear model. In simple words, within each eQTL analysis, we tested each marker for an association with a single gene's expression, resulting in an LOD curve. In order to find out whether the highest LOD value is significant, we performed a permutation analysis with 10 000 permutations and all significant peaks (*α* ≤ 0.001) (Lystig, 2003; Broman *et al*., 2019; Broman, 2023). This process was repeated for all (37 782) active genes. To subsequently also correct for the testing of multiple genes, we considered genes with FDR ≤ 0.001 significant. We performed this procedure on all three cross‐type datasets (IBM‐RIL, B73 × IBM‐RIL, Mo17 × IBM‐RIL). We combined the resulting eQTL peaks, and distinct eQTL were selected. In cases where multiple eQTL were identified for a gene, we assessed whether the different peak positions corresponded to different regulatory elements (≥ 25 Mbp apart or on different chromosomes, and not within the confidence intervals of each other). If multiple eQTL for the same gene did not differ by the specified standards, we only retained the eQTL with the shortest confidence interval or the highest LOD in case of equal confidence intervals. We categorized the eQTL into *cis* and *trans* eQTL based on their distance from the start of their respective gene. We defined *trans‐*regulating eQTL as located at a distance of at least 2.5 Mbp from the start of the gene and where their confidence interval did not include the start of the gene. We classified *cis‐*regulating eQTL as located in proximity to the start of the gene (< 2.5 Mbp) or located such that their confidence interval includes the start of the gene (Pitz *et al*., 2024). This value was initially chosen under the consideration of the average distance of 0.3 Mbp between cross‐overs accumulated over all IBM‐RILs. In addition, the median length of confidence intervals around eQTL was 2.48 Mbp. Investigating the results after this classification, the majority (83%) of *trans*‐regulating eQTL were located on a different chromosome than the corresponding gene. In addition, the majority of *cis*‐regulated genes were located outside of the confidence interval of the eQTL (88%).

## Results

### Transcriptome analysis of two maize intermated recombinant inbred line backcross populations

To study the regulation of nonadditive gene expression in maize hybrids, defined as patterns that significantly deviate from the average of the parental values (MPV), we generated two partially heterozygous backcross populations by crossing 112 IBM‐RIL Syn. 4 RILs to the maternal IBM‐RIL parents B73 and Mo17 (Fig. S1A). The backcross populations, obtained by crossing the IBM‐RILs to their original parents, vary in their heterozygosity as well as heterosis (Fig. S1A).

We subjected 1‐wk‐old primary roots of these backcross hybrids, their parents (IBM‐RILs, B73, or Mo17) and the fully heterozygous hybrids B73 × Mo17 and Mo17 × B73 to RNA‐sequencing and root phenotyping. After quality assessment, 2–3 biological replicates of 85 B73 × IBM‐RIL and 82 Mo17 × IBM‐RIL backcross hybrids were retained for downstream analyses. To obtain higher accuracy in the pairwise comparisons, we included more replicates of the fully heterozygous reciprocal hybrids B73 × Mo17 (23 biological replicates) and Mo17 × B73 (24 biological replicates), and the parental inbred lines B73 (47 biological replicates) and Mo17 (42 biological replicates) in our analyses (Fig. S1B).

### Most nonadditively expressed genes are expressed above the mid‐parent value in hybrids

Nonadditively expressed genes in hybrids are expressed significantly higher or lower than the MPV. To study nonadditive gene expression, we determined genes with significantly different expression in the hybrid compared to the MPV (FDR ≤ 0.05, |Log_2_FC| > 1).

**Fig. 1:**
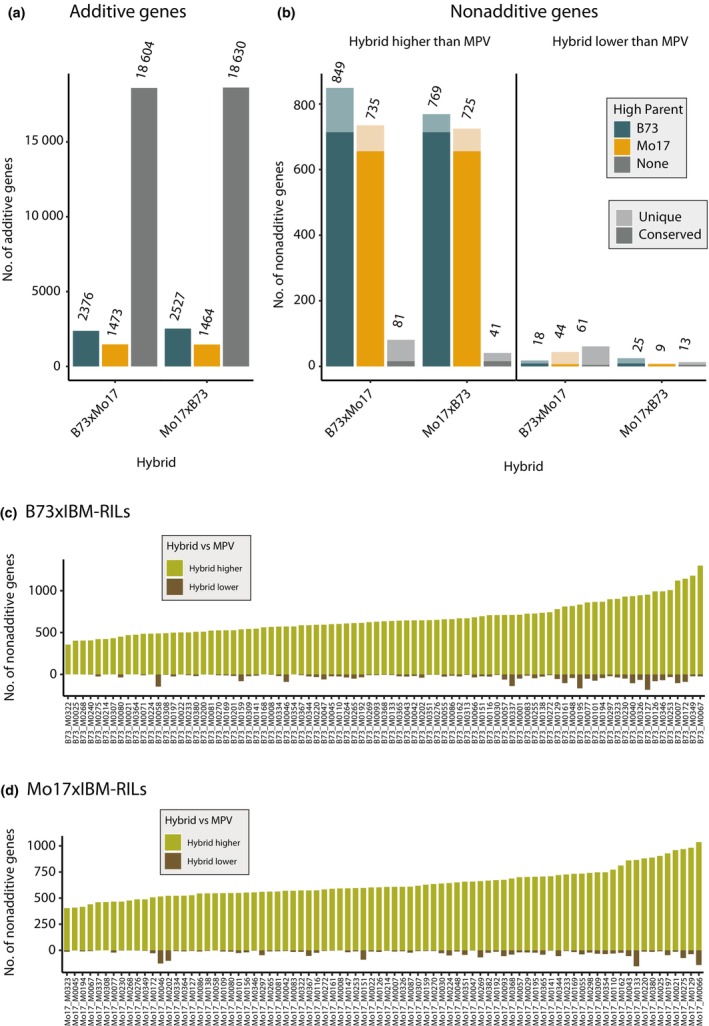
Distribution of gene expression pattern in *Zea mays* L. (a) Numbers of additive genes (difference between mid‐parent value of gene expression (MPV) and hybrid not significant, |Log_2_FoldChange| > 1, *P* < 0.05) in B73 × Mo17 and Mo17 × B73 hybrids. (b) Numbers of nonadditive genes with either significantly higher‐than‐MPV expression or lower‐than‐MPV expression in the hybrid (|Log_2_FoldChange| > 1, *P* < 0.05). Colors in (a) and (b) indicate parental gene expression difference, blue – higher expression in B73 than Mo17 (|Log_2_FoldChange| > 1, *P* < 0.05), yellow – higher expression in Mo17 than B73 and gray – no significant difference. In (b) darker shade indicates conserved expression between the reciprocal hybrids. (c) Numbers of nonadditive genes in B73 × IBM‐RIL and (d) Mo17 × IBM‐RIL hybrids. Different colors indicate whether the hybrid expression value is significantly (|Log_2_FoldChange|> 1, *P* < 0.05) higher (gray, positive values) or lower (brown, negative scale) than the MPV.

In the parent‐hybrid triplets of the fully heterozygous reference hybrids, we investigated 24 241 (B73 × Mo17) and 24 203 (Mo17 × B73) genes active in at least one genotype. Among those, 22 453 (93%; B73 × Mo17) and 22 621 (93%; Mo17 × B73) were additively expressed, of which 83% (B73 × Mo17: 18 604) and 82% (Mo17 × B73: 18 630) did not show any expression difference between the parents (Fig. 1a). The remaining additively expressed genes adopted the MPV of their differentially expressed parents with either B73 or Mo17 being the high parent (Fig. 1a). Among the 1788 (B73 × Mo17) and 1582 (Mo17 × B73) nonadditively expressed genes (Fig. 1b), 93% (B73 × Mo17) and 97% (Mo17 × B73) showed a higher expression level than the MPV (Fig. 1b). Most of these genes (1584 (95%) in B73 × xMo17 and 1494 (93%) in Mo17 × B73) showed significantly different expression between the parents (DEGs: FDR ≤ 0.05, |Log_2_FC| > 1) (Fig. 1b). B73 was the high parent in 54% (B73 × Mo17) and 51% (Mo17 × B73) of genes with differentially expressed parents in the highly heterozygous reference hybrids (Fig. 1b ‘Hybrid higher than MPV’, Dataset S2). Among those, 79% (714) of genes with B73 as the high parent and 82% (656) of genes with Mo17 as the high parent were conserved between the reciprocal reference hybrids (Fig. 1b; Dataset S2). Nevertheless, the expression of 94% (B73 × Mo17) and 98% (Mo17 × B73) of these genes was within the range of their parents.

The average numbers of nonadditively expressed genes among the backcross populations in general were 847 in B73 × IBM‐RILs and 807 in Mo17 × IBM‐RILs, which is approximately half the number (47 and 51%) of nonadditive genes in the fully heterozygous B73 × Mo17 and Mo17 × B73 hybrids. For further analyses, the active genes in the IBM‐RIL backcross populations were classified into heterozygous and homozygous, based on SNPs present in these genes or surrounding regions. Among those, we identified on average 19 042 additive genes in B73 × IBM‐RIL backcrosses and 18 985 in Mo17 × IBM‐RIL backcrosses (Dataset S3). For most nonadditively expressed genes, we observed a prevalence for higher expression compared to the MPV across all B73 × IBM‐RILs (Fig. 1c) and Mo17 × IBM‐RILs (Fig. 1d). In B73 × IBM‐RILs, on average 668 (95%) genes were expressed higher than the MPV (Fig. 1c), while in Mo17 × IBM‐RILs, 637 (96%) displayed this expression pattern (Fig. 1d). By contrast, only 35 (5%; B73 × IBM‐RIL) and 26 (4%; Mo17 × IBM‐RIL) genes were expressed lower in the hybrid compared to the MPV. Hence, the trend of nonadditively expressed genes to exceed the MPV, as observed in the reference hybrids (Fig. 1b), is also conserved in both IBM‐RIL backcross populations (Fig. 1c,d). We therefore focused our downstream analyses on nonadditively expressed genes displaying above‐MPV expression in the hybrids, with parents displaying contrasting expression.

### Heterozygosity drives nonadditive gene expression in backcross hybrids

**Fig. 2:**
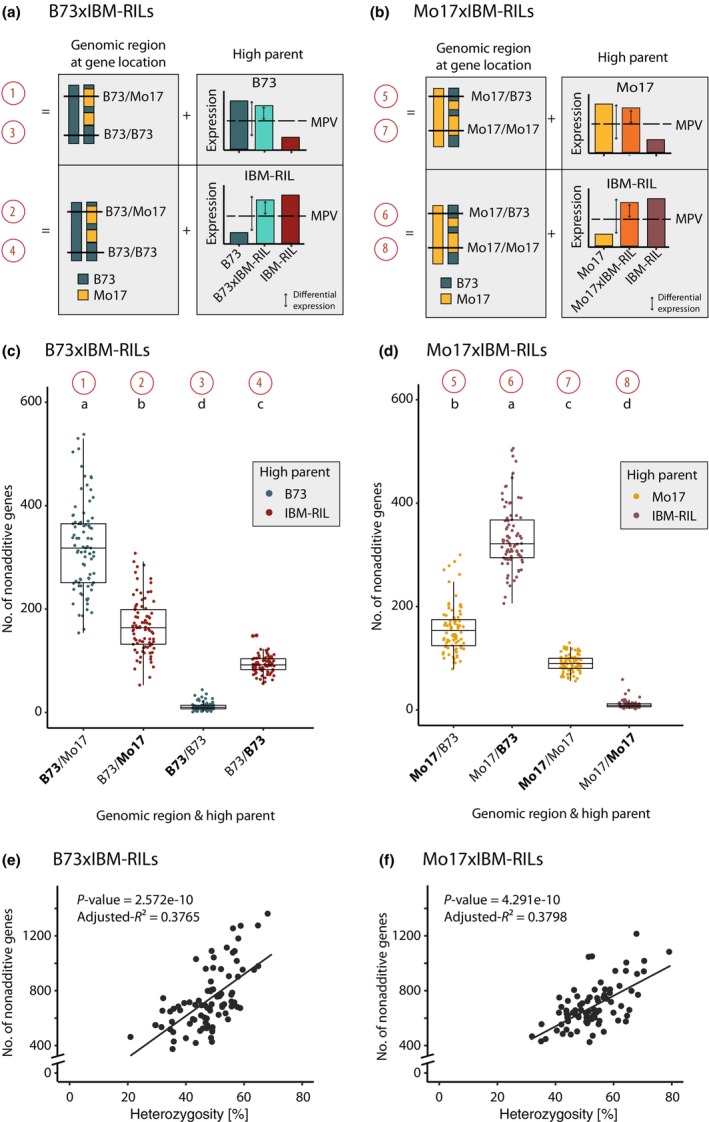
Heterozygous genomic regions drive nonadditive gene expression in backcross hybrids in *Zea mays* L. (a) Schematic depiction of the genomic region of a gene in the hybrid and the possible expression pattern with a higher than mid‐parental value (MPV) in B73 × IBM‐RILs and (b) Mo17 × IBM‐RILs. (c) Numbers of nonadditive genes in B73 × IBM‐RIL hybrids differentiated by expression pattern. (d) Numbers of nonadditive genes in Mo17 × IBM‐RIL hybrids differentiated by expression pattern. Colors in (c) and (d) indicate the parent with higher expression. The high parent is also indicated on the x‐axis in bold letters. The red numbers correspond to the graphical description in (a) and (b). The boxes of boxplots indicate the 25% and 75% quantiles, and the horizontal line indicates the median. The whiskers extend to the largest and smallest value no further than 1.5 times the interquartile range. Different lowercase letters indicate significantly different numbers of nonadditive genes (*α* < 0.05) identified with a Gaussian mixed model with the hybrid as the random effect, the expression patterns as the fixed factor, and a diagonal variance component for the expression pattern. For (e) B73 × IBM‐RIL hybrids and (f) Mo17 × IBM‐RIL hybrids, the heterozygosity of each hybrid in % of all classified regions is displayed against the corresponding number of nonadditive genes. The *P*‐value and adjusted *R* ^2^ are indicated for a linear regression with the heterozygosity as independent and the number of nonadditive genes as the dependent variable.

In the B73 × IBM‐RIL and Mo17 × IBM‐RIL backcross hybrids, nonadditively expressed genes are located in either homo‐ or heterozygous regions of the genome (Fig. 2a,b). In B73 × IBM‐RIL hybrids, most nonadditively expressed genes with higher than MPV expression (on average 487, 82%) were located in heterozygous genomic regions (B73/Mo17, Fig. 2c, patterns 1 and 2), while fewer genes (on average 105, 18%) were observed in homozygous genomic regions (B73/B73, patterns 3 and 4) (Fig. 2c). In the homozygous B73/B73 regions of B73 × IBM‐RILs backcross hybrids, we almost exclusively observed nonadditive genes with the paternal IBM‐RILs as the high parent (90%, Fig. 2c, pattern 4 vs pattern 3).

Similarly, in Mo17 × IBM‐RILs, nonadditively expressed genes with higher expression levels than the MPV were preferentially (on average 492; 83%) detected in heterozygous genomic regions (Mo17/B73, patterns 5 and 6); while fewer genes (101; 17%) were detected in homozygous genomic regions (Mo17/Mo17, patterns 7 and 8) (Fig. 2d). In homozygous regions, we almost exclusively observed nonadditive genes with the maternal high parent Mo17 in Mo17 × IBM‐RILs (90%; Fig. 2c, pattern 7 vs pattern 8).

In heterozygous regions of backcross hybrids, we observed more genes with B73 as the high parent (66% in B73 × IBM‐RILs; Fig. 2c pattern 1 vs pattern 2, 69% in Mo17 × IBM‐RILs; Fig. 2d pattern 5 vs pattern 6). It should be noted that in the fully heterozygous reference hybrids of B73 and Mo17, only 51% and 54% of nonadditive genes had B73 as the high parent (Fig. 1b). The slightly higher number of B73 high parent genes in the reference hybrids (Fig. 1b) might be due to a minor mapping bias or biological dominance of B73. The larger differences within the backcrosses are likely caused by the distinct regulation of B73 and Mo17 high parent genes. In a fully heterozygous genome, patterns 4 and 7 would likely result in the Mo17 high parent pattern because the active alleles in these patterns would be Mo17 (Fig. 2c,d). This would reduce the allelic difference to a similar degree as observed in Fig. 1b.

In summary, in both backcross hybrid populations, B73 × IBM‐RIL and Mo17 × IBM‐RIL, nonadditive genes with higher‐than‐MPV expression were predominantly located in heterozygous regions. Furthermore, the degree of heterozygosity in the backcross hybrids was significantly positively associated with the number of nonadditive genes (Fig. 2e,f), suggesting that heterozygosity is a major driver of nonadditive gene expression.

### Up to 27% of heterotic variance in root traits can be explained by nonadditive genes in Mo17 × IBM‐RILs

We further used a linear modeling approach to calculate the proportion of heterotic variance that can be attributed to the number of nonadditively expressed genes in patterns 1–8 (Table 1). We determined that up to 27% of heterotic variance (variance in hybrid phenotypes that is not accounted for by experimental factors or parental values) can be attributed to nonadditively expressed genes (Table 1). The different traits in the different populations show contrasting values in the heterotic variance attributable to nonadditive genes. This indicates an important but variable role of nonadditive genes for heterosis of different traits and populations (Table 1).

**Table 1:** Proportion of heterotic variance explained by the number of nonadditive pattern 1–8 genes on mid‐parent heterosis for different root phenotypes in *Zea mays* L.

Trait	B73 × IBM‐RILs			Mo17 × IBM‐RILs		
	*σ* ^2^_Het_	*σ* ^2^_G_	*p*_Het_	*σ* ^2^_Het_	*σ* ^2^_G_	*p*_Het_
No. of root tips	0.277	0.332	17% (0.167)	0.155	0.194	20% (0.198)
Total root volume	2.498	2.705	8% (0.077)	0.817	1.123	27% (0.273)
Total root length	3.094	3.567	13% (0.133)	1.786	2.194	19% (0.186)
Lateral root density	5.407	6.805	21% (0.206)	7.366	6.925	‐7% (−0.063)

*σ* ^2^_Het_, unexplained genetic variance of heterosis effect, not associated with nonadditive genes; *σ* ^2^_G_, total genetic variance among the hybrid genotypes; *p*_Het_, coefficient of determination: proportion of the heterotic variance explained by the number of nonadditive genes. [Correction added on 23 May 2025, after first online publication: following a correction to the model used in this work, some of the values in this table have been updated.]

### Nonadditive genes with Mo17 as high parent are predominantly regulated in *trans*

**Fig. 3:**
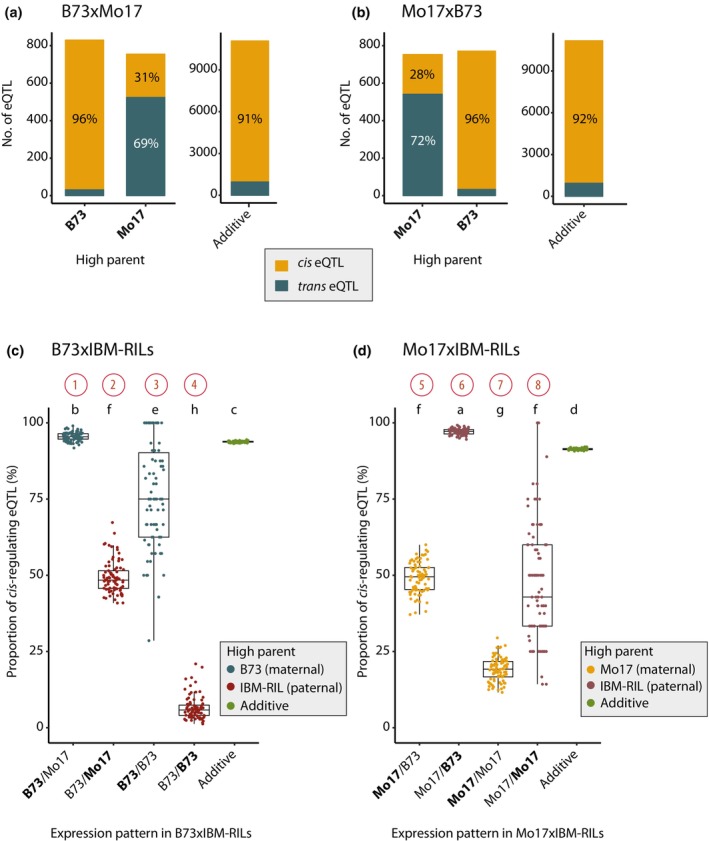
Proportion of *cis*‐ and *trans*‐regulation in expression pattern of nonadditive and additive genes in *Zea mays* L. (a) Bars show the number of *cis* and *trans* regulating expression quantitative trait loci (eQTL) of the genes with different expression patterns in the B73 × Mo17 hybrid and (b) the Mo17 × B73 hybrid. Percentages indicate the proportion of *cis*‐ or *trans*‐regulation. Boxplots display the proportion of *cis*‐regulation among nonadditive expression patterns and additive genes in (c) B73 × IBM‐RIL and (d) Mo17 × IBM‐RIL hybrids. Different lowercase letters indicate significantly different proportions (*α* < 0.05), identified with a Gaussian mixed model with the hybrid as a random effect, the nonadditive expression patterns and additive genes as a fixed factor, and a diagonal variance component for the fixed factors. The boxes of boxplots indicate the 25% and 75% quantiles, the horizontal line the median. The whiskers extend to the largest/smallest value no further than 1.5 times the interquartile range.

To study the regulation of nonadditively expressed genes, we identified eQTL that are significantly associated with the expression of these genes and are thus likely to regulate their activity. For 93% of the nonadditively expressed genes in B73 × Mo17 and for 96% of these genes in Mo17 × B73, we identified at least one eQTL (Dataset S2). We further distinguished the eQTL into *cis*‐regulating in the case of close proximity to the regulated gene (< 2.5 Mbp) and *trans*‐regulating in the case of distal regulation (> 2.5 Mbp, in most instances on a different chromosome). In both reference hybrids, we observed that when B73 was the high parent, genes were primarily *cis*‐regulated by eQTL (Fig. 3a, B73 × Mo17: 96%; Fig. 3b, Mo17 × B73: 96%) while, when Mo17 was the high parent, genes were preferentially *trans‐*regulated by eQTL in the reciprocal hybrids (Fig. 3a, B73 × Mo17: 69%; Fig. 3b, Mo17 × B73: 72%).

For IBM‐RIL backcross hybrids, we identified eQTL for 90% (633/699) of nonadditively expressed genes with higher‐than‐MPV expression on average. In heterozygous regions of B73 × IBM‐RIL and Mo17 × IBM‐RIL hybrids, we observed a similar pattern as in the fully heterozygous B73 × Mo17 and Mo17 × B73 hybrids: genes with the high parent contributing the B73 allele were almost exclusively controlled by *cis*‐regulating eQTL (Fig. 3c, pattern 1, B73 × IBM‐RILs: 96%; Fig. 3d, pattern 6, Mo17 × IBM‐RILs: 97%), whereas genes with the Mo17 allele as high parent showed a similar number of *cis*‐ and *trans*‐regulating eQTL (Fig. 3c, pattern 2, B73 × IBM‐RILs: 49% *cis*; Fig. 3d, pattern 5, Mo17 × IBM‐RILs: 49% *cis*). Among additive genes, on average 94% and 91% were *cis*‐regulated (Fig. 3c,d).

### Heterozygous eQTL regulate heterozygous and homozygous nonadditive genes

**Fig. 4:**
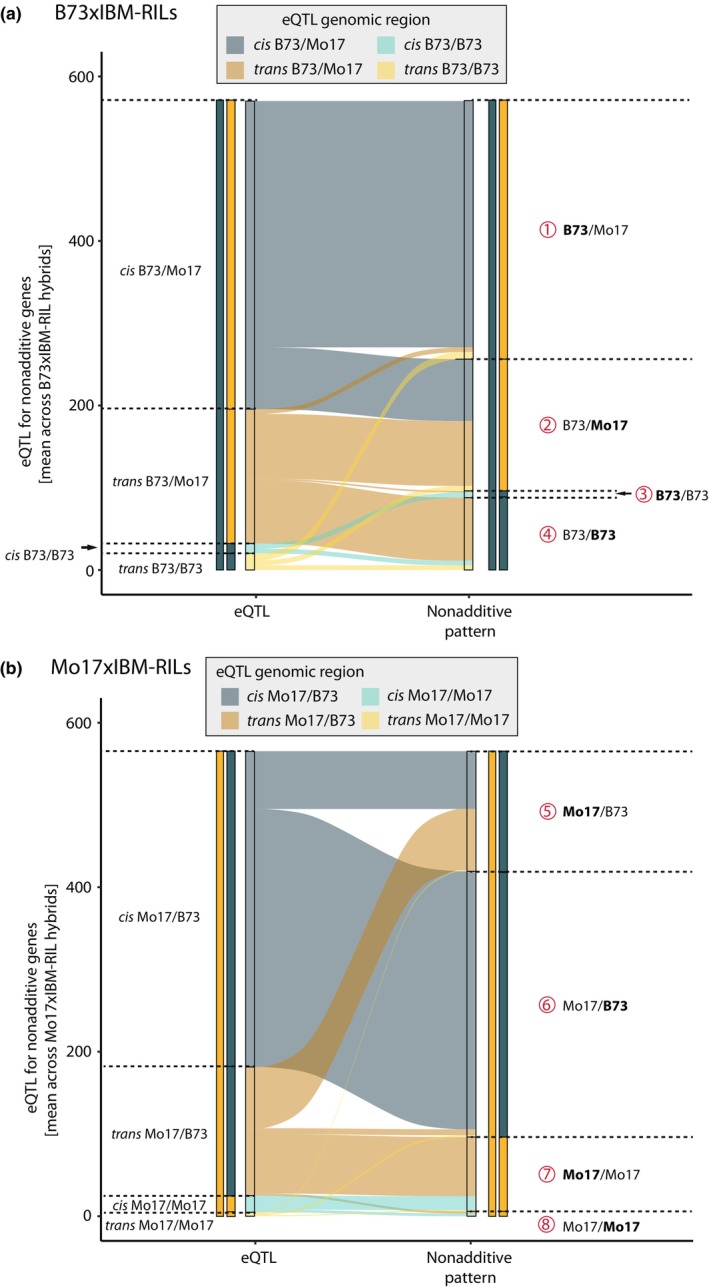
The distribution and location of expression quantitative trait loci (eQTL) regulating nonadditive genes in homo‐ or heterozygous regions in *Zea mays* L. (a) Distribution in B73 × IBM‐RIL hybrids and (b) Mo17 × IBM‐RIL hybrids. Nonadditive genes can be expressed higher (|Log_2_FoldChange| > 1, *P* < 0.05) in the maternal parent or the paternal parent (indicated in bold on the right‐hand side). Corresponding eQTL can be *cis*‐regulating from heterozygous (dark blue) or homozygous (light blue) regions or *trans*‐regulating from heterozygous (dark yellow) or homozygous (light yellow) regions, additionally written on the left‐hand side.

**Fig. 5:**
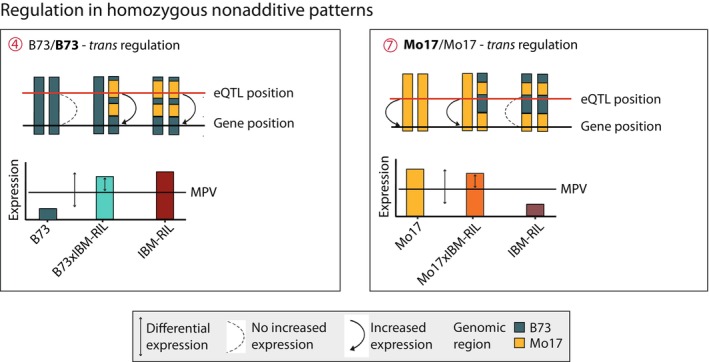
Schematic depiction of the *trans*‐regulation of nonadditive genes in homozygous regions in *Zea mays* L. Expression is initiated by the Mo17 allele at the expression quantitative trait locus (eQTL) position in both cases. Mo17 genomic regions are indicated in yellow, and B73 genomic regions are indicated in blue.

Next, we distinguished between heterozygous and homozygous eQTL in the backcross hybrids. Since *cis*‐acting eQTL are located in close proximity to their target gene, we observed that *cis*‐acting eQTL generally share the same zygosity (i.e. homo‐ or heterozygote) as the nonadditive gene they regulate (Fig. 4a,b). Thus, most *cis*‐acting eQTL regulating nonadditive genes are located in heterozygous regions for B73 × IBM‐RILs (Figs 4a, S2A) and Mo17 × IBM‐RILs (Figs 4b, S2B) because most nonadditively expressed genes are located in heterozygous genomic regions (Fig. 2c,d). *Trans*‐acting eQTL are also almost exclusively located in heterozygous regions, but they regulate heterozygous and homozygous nonadditively expressed genes at a similar rate (Fig. 4a, patterns 2 and 4, Fig. 4b, patterns 5 and 7). Thus, nonadditive genes with expression patterns 1–8 (Fig. 2) are almost exclusively regulated in heterozygous regions (B73 × IBM‐RILs: 94%, Mo17 × IBM‐RILs: 95%). In the homozygous regions in B73 × IBM‐RILs, nearly all nonadditive genes displayed pattern 4 (Fig. 2c) and eQTL for those genes were predominantly *trans*‐regulating (94%; Fig. 3a). These eQTL were located almost exclusively in the heterozygous regions (Fig. 4a, pattern 4). This indicates that, for pattern 4, although these genes were homozygous for the B73 allele, they were regulated by eQTL that also carried the Mo17 allele at the heterozygous location of the eQTL (Fig. 5, pattern 4). Thus, genes with the same allele in both parents display differential expression. In this case, the IBM‐RIL parent providing the Mo17 allele at the eQTL position was highly expressed. By contrast, genes in homozygous regions in Mo17 × IBM‐RILs had the maternal Mo17 as the high parent (pattern 7) but were also *trans*‐regulated (81% *trans*‐regulated; Fig. 3b). In this pattern, the genes were located in homozygous Mo17/Mo17 regions, while the eQTL were located in heterozygous Mo17/B73 regions (Fig. 4b, pattern 7) with the IBM‐RIL providing the B73 allele at the eQTL. At the same time, the expression (as per definition of the pattern 7) was lower in the IBM‐RIL than in the Mo17 parental line (Fig. 5, pattern 7).

In summary, nonadditive genes in heterozygous regions with the Mo17 allele provided by the high parent are more often *trans*‐regulated compared with those with B73 as the high parent. We further demonstrated that nonadditive genes in homozygous regions are regulated by *trans*‐eQTL from heterozygous regions, where the Mo17 allele at the eQTL is responsible for increased gene expression.

## Discussion

The dominance model of heterosis suggests that deleterious alleles are complemented by beneficial alleles at many loci in the hybrid (Jones, 1917), while the overdominance model explains heterosis by beneficial interactions of alleles in heterozygous genes in the hybrid (Shull, 1908). It has been suggested that heterosis is controlled by several genetic mechanisms, with varying contributions depending on the species and traits under analysis (Schnable & Springer, 2013). Results of transcriptome studies supported the notion that specific gene expression patterns can contribute to heterosis, although no direct correlation between differential expression or nonadditive expression and heterosis has been identified before (Hochholdinger & Baldauf, 2018). Here, we demonstrated how nonadditively expressed genes and their regulation contribute to heterosis. For this purpose, we analyzed gene expression profiles of the fully heterozygous reference hybrids B73 × Mo17 and Mo17 × B73 and populations of partially heterozygous B73 × IBM‐RIL and Mo17 × IBM‐RIL hybrids.

In general, we observed that most genes in fully and partially heterozygous hybrids were additively expressed, suggesting their expression is not different from the MPV. This is in line with previous studies where, in general, > 90% of genes in maize hybrids were additively expressed (Stupar & Springer, 2006; Paschold *et al*., 2012). This maintenance of the *status quo* for most genes was suggested to be beneficial for the hybrid (Stupar & Springer, 2006) and is in line with the gene balance hypothesis (Birchler & Veitia, 2010), which states that quantitative traits are influenced by gene dosages of different alleles of different genes (Schnable & Springer, 2013; Yao *et al*., 2013). The number of nonadditively expressed genes in the reference crosses in the present study is consistent with previous observations (Baldauf *et al*., 2020). As expected, in the backcross hybrids we observed only approximately half the number of nonadditively expressed genes compared to the reference crosses because they are predominantly observed in heterozygous regions of the genome. We demonstrated that most nonadditive genes were expressed higher than mid‐parental expression. This pattern was universal across all backcross and reference hybrids. Previous studies showed a similar pattern, in which more nonadditive genes adopted the high‐parent expression in the same maize (Paschold *et al*., 2012), cotton (Yoo *et al*., 2013) or coffee (Combes *et al*., 2015) hybrids. Recently, a thermodynamic model of transcription factor binding in hybrids suggested this to be a general mechanism in cases of parental expression divergence, based on above‐average occupancy of promoters (Janko *et al*., 2024). This trend of expression complementation is consistent with the dominance model of heterosis (Jones, 1917). Similar observations were made in *Arabidopsis*, where different pathways were complemented in a high‐parent expression pattern, which was connected to hybrid adaptability across developmental periods (Liu *et al*., 2021).

**Fig. 6:**
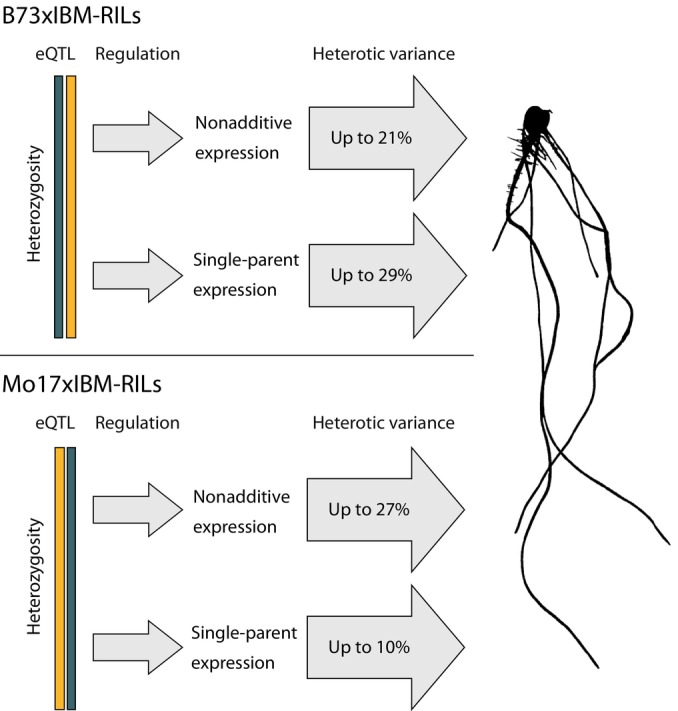
Contribution of nonadditive (this study) and single‐parent expression (SPE) complementation (Pitz *et al*., 2024) to heterotic variance of root traits in *Zea mays* L. Both nonadditive and SPE complementation in B73 × IBM‐RIL and Mo17 × IBM‐RIL backcross populations are predominantly regulated by qtl (expression quantitative trait loci (eQTL)) in heterozygous regions of the genome.

We demonstrated that up to 27% of heterotic variance in phenotypic root traits can be explained by the number of nonadditively expressed genes in Mo17 × IBM‐RILs (Table 1). Similarly, genes displaying SPE, a pattern where a gene is expressed in the hybrid but in only one of the parents, influenced heterosis significantly up to 29% in the backcross population B73 × IBM‐RILs (Pitz *et al*., 2024). Remarkably, while SPE genes contributed mainly in B73 × IBM‐RIL backcross hybrids to heterosis, nonadditively expressed genes contributed substantially to heterosis in both Mo17 × IBM‐RIL and B73 × IBM‐RIL backcross hybrids, with the exception of lateral root density (Pitz *et al*., 2024) (Fig. 6). Thus, nonadditive genes and SPE genes appear to contribute to heterosis in a genotype‐ and trait‐specific manner, with variable contribution.

Using IBM‐RIL backcross populations allows to study the regulation of nonadditively expressed genes via eQTL analyses. Additionally, the partially hetero‐ and homozygous nature of our backcross populations revealed aspects of nonadditive gene expression regulation that cannot be studied in fully heterozygous hybrids. In both IBM‐RIL backcross hybrid populations, eQTL regulating nonadditive gene expression were almost exclusively located in heterozygous regions (B73 × IBM‐RILs: 94%, Mo17 × IBM‐RILs: 95%), regardless of which allele (B73/Mo17) was contributed by the higher expressed parent. This is in line with the observed numbers of nonadditive genes. In the partially homozygous backcross populations, about half as many nonadditive genes were identified compared to the fully heterozygous hybrids. On average, only *c*. 50% of the genome of the backcross hybrids is heterozygous and nonadditive expression is regulated in those heterozygous regions. While SPE complementation (Baldauf *et al*., 2018, 2022) and nonadditive expression are consistent with the dominance model, these expression patterns are regulated by heterozygous eQTL interactions, suggesting overdominance (Pitz *et al*., 2024). These observations are consistent with the notion that the dominance and overdominance models are not mutually exclusive (Schnable & Springer, 2013). In our study, dominance prevailed at the level of gene expression, while overdominance was observed predominantly at the level of gene regulation.

A possible explanation for the observed differences in the contribution of heterozygosity and nonadditive expression between the different backcross populations might be related to the different regulation modes of nonadditive expression patterns: We discovered that nonadditive genes with Mo17 as the high parent were predominantly *trans*‐regulated (*c*. 70%), while those with B73 as the high parent were almost exclusively *cis*‐regulated (*c*. 95%) (Fig. 3a,b). In a previous study, *trans*‐regulation was associated with paternal dominance (Swanson‐Wagner *et al*., 2009). Although Mo17 is traditionally the paternal parent, we observed that *trans*‐regulation associated with Mo17 expression was independent of the maternal or paternal origin. A similar observation was made for genes that display SPE, where the expression of a gene in only one parent is complemented in the hybrid (Pitz *et al*., 2024). In summary, Mo17 dominance, in terms of higher expression or activity, is largely *trans*‐regulated, while B73 dominance is preferentially *cis*‐regulated. The same tendency was observed for SPE genes with either Mo17 (*trans*‐regulation) or B73 (*cis*‐regulation) as the active parent (Pitz *et al*., 2024). Based on our findings, we suggest that one of the factors contributing to the outstanding hybrid performance of B73 and Mo17 hybrids might be the distinct regulation (*cis* vs *trans*) of nonadditive and SPE genes of these genotypes. The two inbred lines B73 and Mo17 had contrasting breeding objectives and originated from different breeding pools. B73 belongs to the Iowa stiff stalk synthetic/BSSS group, typically used as a female parent, while Mo17 belongs to the Lancaster group and is a typical male parent (Troyer, 2001). Those differences might have unintentionally resulted in the observed differences in regulation. Differences in regulation likely increase the possibilities for transcriptional activity in the resulting hybrids. It was previously shown that commercial maize inbred lines of the B73 heterotic subgroup were likely selected toward the B73 founder alleles (White *et al*., 2020). It is therefore likely that in addition to phenotypic traits, regulatory preferences were passed on within a heterotic subgroup as well, influencing contemporary hybrid maize breeding pools. The observed combination of *cis*‐ and *trans*‐regulated gene expression results in a complementation of higher than mid‐parental gene expression. Due to their conservation under different conditions, nonadditive expression was proposed to be beneficial to the hybrid under different environmental conditions (Marcon *et al*., 2017). Additionally, complementation of gene expression and function during plant development was suggested to contribute to heterosis in Arabidopsis (Liu *et al*., 2021). The presented differences in the high parent regulation regime might explain the different results obtained for the two different populations, as not all genes can be complemented in the backcross hybrids.

While higher expression is not necessarily beneficial for every phenotypic trait and for every gene and condition, nonadditive gene expression was suggested to be beneficial for hybrids to thrive under different environmental cues (Marcon *et al*., 2017; Liu *et al*., 2021). We demonstrated that eQTL associated with nonadditively expressed genes are mainly located in heterozygous regions, leading to a complementation of higher than mid‐parent expression across nonadditive genes, showing how heterozygosity at the regulatory level influences complementation of gene expression. We further showed that genes displaying nonadditive expression patterns contribute to heterosis and that their regulation might be a new aspect necessary to translate phylogenetic distance into vigorous hybrids. Based on our results, we hypothesize that diverging regulatory preferences in inbred lines are beneficial for selecting parental combinations for hybrid breeding. In future research, the regulatory preferences for nonadditive and SPE genes could be tested for parental breeding pools and their hybrid combinations other than B73 and Mo17.

## Competing interests

None declared.

## Author contributions

MP and JAB carried out the experiments, conducted the statistical analyses, interpreted the data, and drafted the manuscript. H‐PP provided help with the experimental design for the RNA‐seq experiment and helped with the statistical analyses. FH conceived and coordinated the study and participated in data interpretation and drafting the manuscript. All authors edited the manuscript and approved the final draft. MP and JAB contributed equally to this work.

## Disclaimer

The New Phytologist Foundation remains neutral with regard to jurisdictional claims in maps and in any institutional affiliations.

## Supporting information


**Dataset S1** Proportions of heterozygous regions in the hybrids.
**Dataset S2** Details on nonadditive genes in the reference hybrids of B73 and Mo17.
**Dataset S3** Summaries of additive and nonadditive genes in all hybrids.

**Fig. S1** Investigated parent and hybrid genotypes. Modified after Pitz *et al*. (2024).
**Fig. S2** Boxplots showing expression quantitative trait loci (eQTL) of nonadditive genes.
Please note: Wiley is not responsible for the content or functionality of any Supporting Information supplied by the authors. Any queries (other than missing material) should be directed to the *New Phytologist* Central Office.

## Data Availability

The data is set to be released upon publication NCBI Bioproject ID PRJNA923128 (https://www.ncbi.nlm.nih.gov/bioproject/PRJNA923128).
