# Supplementary material for: Nonadditive gene expression contributing to heterosis in partially heterozygous maize hybrids is predominantly regulated from heterozygous regions
Source: New Phytol. 2025 Apr 7;247(2):669–83. doi: 10.1111/nph.70128 (PMC12177283; doi:10.1111/nph.70128)
Supplement: Supplementary file 2 — Fig. S1 Investigated parent and hybrid genotypes. Modified after Pitz et al. (2024). Fig. S2 Boxplots showing expression quantitative trait loci (eQTL) of nonadditive genes. Please note: Wiley is not responsible for the content or functionality of any Supporting Information supplied by the authors. Any queries (other than missing material) should be directed to the New Phytologist Central Office. [file NPH-247-669-s002.docx]

## *New Phytologist* Supporting Information

Article title: Non-additive gene expression contributing to heterosis in partially heterozygous maize hybrids is predominantly regulated from heterozygous regions

Authors: Marion Pitz, Jutta A. Baldauf, Hans-Peter Piepho, Frank Hochholdinger

Article acceptance date: 14 March 2025

The following Supporting Information is available for this article:

**Fig. S1** Investigated parent and hybrid genotypes. Modifed after Pitz *et al.* 2024


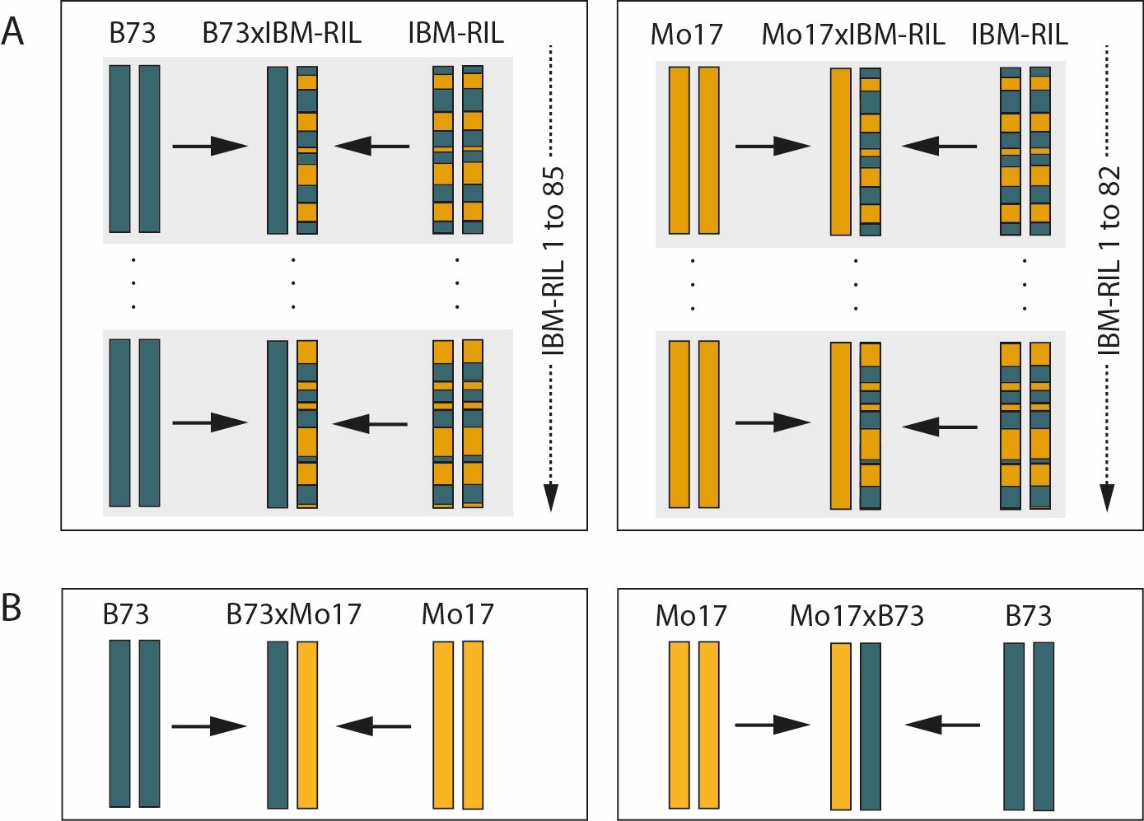


**Fig. S2** Boxplots showing eQTL of non-additive genes. **A** B73xIBM-RIL hybrids or **B** Mo17xIBM-RIL hybrids. Non-additive expression pattern is shown on the x-axis, with the genotype as indicated to distinguish hetero- and homozygous genes and the higher expressed parent (|Log2FC|>1, p<0.05) indicated in bold, or no signifcant di_erence in the parents (No high parent). The associated eQTL can be *cis*-regulating from heterozygous (dark blue) or homozygous (light blue) regions or *trans*-regulating from heterozygous (dark yellow) or homozygous (light yellow) regions.


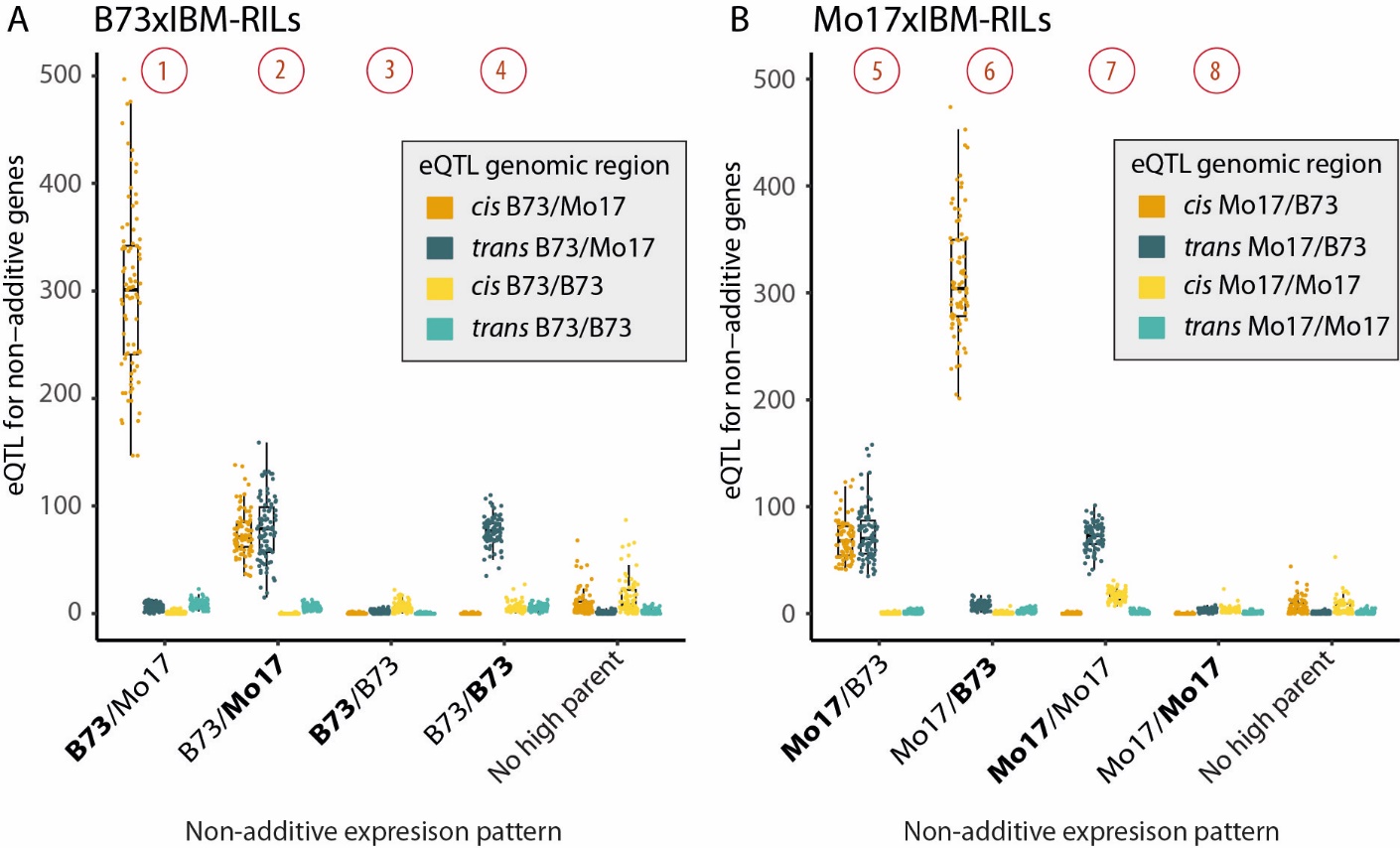


**Provided in separate excel file “Data S1 to S3.xlsx”**

**Data S1** Data_S1_het_prop; Proportions of heterozygous regions in terms of length among all classified regions in the hybrids.

**Data S2** Data_S2_NAG_refs; Non-additive pattern of all genes in reference hybrids. Expression_pattern are defined as NAG_B73/Mo17_M_B73 (B73 as maternal high parent), NAG_B73/Mo17_P_Mo17 (Mo17 as paternal high parent), NAG_Mo17/B73_M_Mo17 (Mo17 as maternal high parent, NAG_Mo17/B73_P_B73 (B73 as paternal high parent), cis_trans indicates the classification of regulating eQTL.

**Data S3** Data_S3_overview; Summarized details of additive and non-additive genes in all hybrids. The numbers of genes with additive expression in the hybrid, higher expression or lower expression in the hybrid are indicated for each hybrid of the B73 and Mo17 backcross populations.

**References**

**Pitz** **M, Baldauf** **J, Piepho** **H-P*, et al.* 2024**. Regulation of heterosis-associated gene expression complementation in maize hybrids. *bioRxiv*: 2024.10.30.620956. doi:10.1101/2024.10.30.620956.
